# An Artificial Neural Network Model for Assessing Frailty-Associated Factors in the Thai Population

**DOI:** 10.3390/ijerph17186808

**Published:** 2020-09-18

**Authors:** Nawapong Chumha, Sujitra Funsueb, Sila Kittiwachana, Pimonpan Rattanapattanakul, Peerasak Lerttrakarnnon

**Affiliations:** 1 Aging and Aging Palliative Care Research Cluster, Faculty of Medicine, Chiang Mai University, Chiang Mai 50200, Thailand; nawapong_c@cmu.ac.th; 2 Department of Chemistry, Faculty of Science, Chiang Mai University, Chiang Mai 50200, Thailand; sujitra.funs@gmail.com (S.F.); silacmu@gmail.com (S.K.); 3 Geriatric Medical Center, Faculty of Medicine, Chiang Mai University, Chiang Mai 50200, Thailand; myping13@gmail.com; 4 Department of Family Medicine, Faculty of Medicine, Chiang Mai University, Chiang Mai 50200, Thailand

**Keywords:** frailty, elderly, Self-Organization Map

## Abstract

Frailty, one of the major public health problems in the elderly, can result from multiple etiologic factors including biological and physical changes in the body which contribute to the reduction in the function of multiple bodily systems. A diagnosis of frailty can be reached using a variety of frailty assessment tools. In this study, general characteristics and health data were assessed using modified versions of Fried’s Frailty Phenotype (mFFP) and the Frail Non-Disabled (FiND) questionnaire (mFiND) to construct a Self-Organizing Map (SOM). Trained data, composed of the component planes of each variable, were visualized using 2-dimentional hexagonal grid maps. The relationship between the variables and the final SOM was then investigated. The SOM model using the modified FiND questionnaire showed a correct classification rate (%CC) of about 66% rather than the model responded to mFFP models. The SOM Discrimination Index (SOMDI) identified cataracts/glaucoma, age, sex, stroke, polypharmacy, gout, and sufficiency of income, in that order, as the top frailty-associated factors. The SOM model, based on the mFiND questionnaire frailty assessment, is an appropriate tool for assessment of frailty in the Thai elderly. Cataracts/glaucoma, stroke, polypharmacy, and gout are all modifiable early prediction factors of frailty in the Thai elderly.

## 1. Introduction

In 2019, the world population was 7.7 billion, with 9.1% aged over 65. The Thai aging population will increase to 11.7% in 2030 and 19.5% in 2075. Thailand had 69.6 million people in 2019, with 12.4% of the population over 65. That is predicted to rise to 19.6% in 2027 and 29.6% in 2030 [1]. Based on those projections, the world will become an aged society, defined as a population with more than 7% aged over 65, within 18 to 33 years, a shift that will have profound socioeconomic, cultural and political implications which policymakers will need to address in the areas of health care, long-term care, employment, social protection and provision of an age-friendly environment [2]. For the elderly, frailty is one of the major public health problems. The World Health Organization (WHO) characterizes frailty as “a clinically recognizable state in which the ability of older people to cope with every day or acute stressors is compromised by an increased vulnerability brought on by age-associated declines in physiological reserve and function across multiple organ systems [3].” There are multiple etiologic factors leading to frailty, including biological and physical changes in the body which contribute to reduced bodily functions in multiple systems, including energy reserves and the immune system [4,5]. The prevalence of frailty is currently around 15% for adults aged 65 years and over, based on a recent meta-analysis of community-dwelling older Europeans [6]. Risk factors potentially affecting the health of the elderly come with increasing age, e.g., inflammation and infections, reduced body performance and increased physical impairment combined with disability and chronic disease [7,8]. The factors influencing frailty are generally composed of increasing age, low physical activity, malnutrition and adverse mental states such as depression, declining thinking ability and memory. It has been estimated that between a quarter and half of people older than 85 are frail, and these people have a substantial risk of falls, disability [9] and need of long-term care as well as premature death [10,11]. There have been many studies of frailty assessment tools, not only for clinical use but also for use by the general public [12]. Most of those studies have included quantitative statistical analysis, e.g., logistic regression [13,14], linear regression [15], and hazards regression [16,17,18].

Artificial neural networks (ANNs) are biologically inspired computer programs modeled on how the human brain processes information [19,20,21]. They gather knowledge from repeatedly ascertaining patterns and relationships of data and can learn (or be trained) through experience. With ANNs, each artificial neuron receives a set of inputs related to mathematically coefficients (weights), which are used to establish the neural structure which allows the inclusion of a large number of variables [22,23]. To acquire knowledge in a given target application, the inter-unit connections are optimized until the prediction error is minimized and the network reaches a specified level of accuracy. Due to the utility of the ANNs model, it has been widely applied in pattern recognition and universal data mapping [24,25]. In the medicine and health sciences, ANNs are mainly used for decision support systems, prediction, and data visualization. ANNs has recently been used for diagnosing cancer, pulmonary, pleural tuberculosis and hereditary amyloid polyneuropathy [26,27,28], and for image recognition using electrocardiogram (ECG) and computerized axial tomography (CAT) scans [29,30,31].

In 1984, Kohonen et al. [19] developed an unsupervised learning method using ANNs in which the algorithm learns the structure of the data without any additional information, a process called self-organizing mapping (SOM). This method has noticeable important applications in dimension reduction, data clustering and image analysis. The multidimensional data are contemplated through the learning process and displayed as a low-dimension neuron grid map, typically in two dimensions. The neuron map is updated based on the weights of the winner (the nearest grid determined by the Euclidean distance from the input vector) and its neighboring neurons until reaching a topological order that preserves the similarity between their original characteristics. The result, a SOM map, is composed of the component planes of each parameter. Thus, SOM can interpret or explain the relationship among the studied parameters better than the classical methods. This could be a major advantage over the other ANN models behaving themselves as a ‘black box’, where an input data is given for constructing the models [32]. After intensive calculation, the desired information is given back from some output units. However, it is not possible to investigate the structure inside the models.

In Thailand, a few studies have tried to develop assessment tools for the Thai elderly [33,34], but there are, to date, no generally accepted tools for frailty assessment. The frailty assessment tool from Screening/Evaluation of Elderly Manual, Department of Medical Services, Thai Ministry of Public Health (TMPH), [35] was assessed by members of our team (unpublished data) which determined the tool was modified from Fried’s Frailty Phenotype (FFP) [7]. In that tool, there are five criteria, including weight loss of 4.5 kg or more in the past year, feeling exhausted all time, inability to walk by themselves or needing someone to help them walk, a time of > 7 s for a 4.5 m walk, and a feeling of weakness in the hand, arm or leg [35]. Each criterion is scored 0 for a “no” answer and 1 for a “yes” answer. Frailty is diagnosed if there are three or more positive criteria, similar to the FFP system. However, no comprehensive evaluation of the TMPH tool has been published. A previous study based on unpublished data evaluated the validity and reliability of TMPH and the Frail Non-Disabled (FiND) questionnaire, [36] comparing them with the FFP (the gold standard). The TMPH evaluation system includes four questions and one physical measurement, while the FiND evaluation is based only on five questions and no physical measurements, making it less complicated than the TMPH. This study conducted a pilot investigation of frailty-associated factors using previous unpublished FiND questionnaire data in the Thai population to determine if SOM could be a time- and cost-efficient method of visualizing and validating property relationships between materials [37] as a visualization tool for frailty research.

## 2. Methods

This study was approved by the Research Ethics Committee of the Faculty of Medicine, Chiang Mai University (No. 31/2018).

### 2.1. Patients

In this study, the medical records of 251 elderly patients (98 males and 153 females) of the Out-Patient Clinic, Department of Family Medicine, Maharaj Nakorn Chiang Mai Hospital, were included.

### 2.2. Patient Data

Secondary data on those patients were obtained from the study, “Validity and Reliability of the Thai Version of Frailty Assessment Tools in the Elderly, Primary Care Unit, Maharaj Nakorn Chiang Mai Hospital,” by Rattanapattanakul P. and Lerttrakarnnon P. Information on all patients was reviewed through enquiries of their medical records at Maharaj Nakorn Chiang Mai Hospital. The frailty level of the patients was assessed using two frailty assessment tools, modified versions of the FFP (mFFP) and the Frail Non-Disabled (FiND) questionnaire. Those tools were chosen because they both include the same five variables, and both can accommodate the same modified definition of frailty (Table S1).

Fried’s Frailty Phenotype (FFP) [38] assesses five variables, including weight loss, exhaustion, physical activity, walking time and grip strength. A modified version of FFP (mFFP) was considered the gold standard method in this study. Level of physical activity was assessed using a modified SHARE-FI questionnaire [39] which included, e.g., “How often do you engage in activities that require a low or moderate level of energy such as gardening and cleaning the car?” Responses were scored as 1 = “More than a once a week”, 2 = “Once a week”, 3 = “One to three times a month” and 4 = “Hardly ever or never”. A score of 2 or more was considered low physical activity. Based on the combined scores, patients were classified in one of three groups: 0 = “non-frail”, 1-2 = “prefrail” and 3-5 = “frail”.

The Frail Non-Disabled (FiND) questionnaire [36] included five questions, each answered either “yes” (1) or “no” (2). The questions were (A) “Do you have difficulty walking?”, (B) “Do you have difficulty climbing?”, (C) “Have you lost weight?”, (D) “Do you have a problem with exhaustion?”, and (E) “Do you have a high level of physical activity?” If A+B ≥ 1, the patient was classified as “disabled”; if A+B=0 and C+D+E ≥ 1 the patient was classified as “frail” and if A+B+C+D+E = 0 the patient was classified as “robust”. The interpretation of mFiND scores were 0 = “non-frail”, 1-2 = “prefrail” and 3-5 = “frail”. The FiND walking and climbing variables (A and B) were also in FFP, but the FiND variable “grip strength” had no counterpart in FFP.

### 2.3. Data Preprocessing

Before feeding these data into the ANN for processing, it was necessary to preprocess the data to identify manifest and latent variables. In this study, non-continuous data and data with substantially different scales were standardized by subtracting the mean of each variable and dividing that by its standard deviation, so all the variables would have approximately the same scale [40]. All assessments of patient condition were based on Fried’s Frailty Phenotype scores, i.e., patients were assessed as non-frail, pre-frail or frail if their scores were 0, 1-2 or 3-5, respectively. 

### 2.4. Data Analysis

High-dimensional data analysis was conducted by principal component analysis (PCA) using an unsupervised learning algorithm for initial exploration of the data. In this analysis, data were transformed into several principal components (PCs) or linear combinations of the variables while retaining a maximum amount of the original information [41]. With this system, the first few PCs usually retain most of the variation present in all the original variables, and each PC can be interpreted independently. Similarities and differences can be visualized through a score plot of the PCs. Generally, samples with similar properties are placed in a nearby position. 

Following that, the data were analyzed using supervised self-organizing mapping (SSOM), a supervised classification algorithm. The input variables were identified as data matrix (*X*) and class matrix (*Y*). With this system, the data matrix contains the patient’s variables and the class matrix contains the class of each sample. In this case, the non-frail patients (frailty score = 0) were defined as class 1, pre-frail patients (frailty score = 1-2) were defined as class 2, and frail patients (frailty score = 3-5) were defined as class 3. The class matrix of samples in class 1, shown in column 1 of the class matrix, are labeled 1 and the other samples are labeled 0. Samples in classes 2 and 3 are labeled 1 in columns 2 and 3, respectively. 

In order to avoid overfitting of the model, samples were separate into two sets, a train set (auto prediction) and a test set, using the bootstrap method [40]. Two-thirds of the samples were in the train set and the rest were in the test set. All the samples in the train set were used to construct the SOM model, which was then used to predict the class of the sample or the frailty designation (non-frail, pre-frail, frail). 

Some additional variables, i.e., percentage predictive ability (%PA), percentage model stability (%MS) and percentage correctly classified (%CC), were assessed for accuracy, stability, and ability of the analyzed SSOM model. Additionally, the self-organizing map discrimination index (SOMDI) of each variable was used to estimate the influence of the variables in the created SSOM model [40,42].

**Figure 1:**
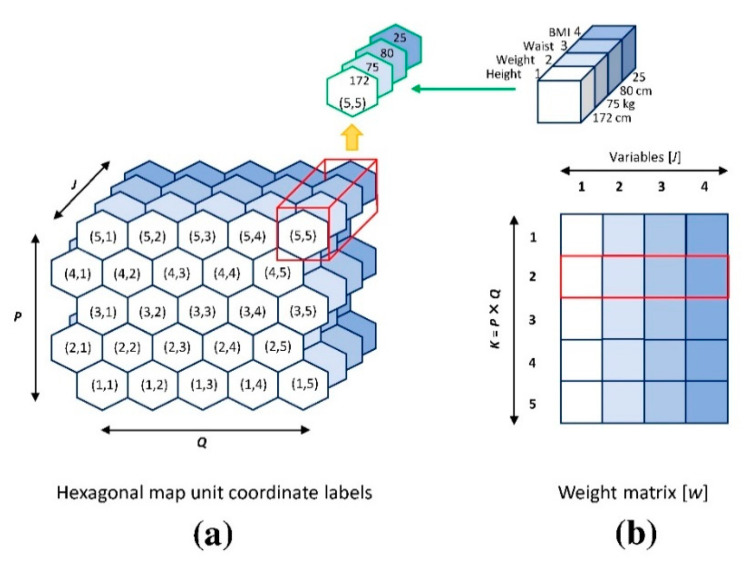
(**a**) Hexagonal map unit coordinate labels and (**b**) weight matrix.

To facilitate data visualization, the SOM was transformed onto a 2-D grid of neurons (in hexagonal form), which were clustered in such a way that possible natural groups of data were evident [43,44]. A hexagonal map unit, composed of the component planes of all 28 variables, was created using random numbers and displayed as minimum to maximum for each parameter (Figure 1a). The weight matrix (Figure 1b) contains all the variables of each sample, representing the neurons in the brain. A data matrix was randomly chosen and compared with all units in the map to find a best matching unit (BMU) based on mathematic calculation (Euclidian distance). The BMU was adjusted to be close to the random sample, and the neighbors of the units were also adjusted based on their similarity to and distance from the BMU. The samples were repeatedly randomized, and the map was adjusted each time (the training protocol) until a stable trained map was obtained. 

Discrimination variables using SSOM can be observed by comparing profiles of the class plane of interest with the component planes of each variable. However, that process requires a very large amount of time and is not feasible for data which include a large number of variables. SOMDI, however, can solve that problem. SOMDI is the score that represents how closely a variable’s component plane corresponds to a class plane of interest [43]. The higher the score, the more important the discriminant variable. In addition, variables which have a positive score vary directly with the class plane of interest, while variables which have a negative score vary inversely with that plane. For data which contained more than two classes, the change in the SOM discrimination index (ΔSOMDI) score, which represents the difference between the class of interest and other classes, was applied. The larger the positive ΔSOMDI value, the greater the ability of the variable to classify the interested class’s samples [44].

The ANN calculations were implemented using in-house MATLAB scripts (MATLAB R2015a, The Mathworks Inc., Natick, MA, USA). Details of SOM algorithms and the parameters used can be found in a paper by Lloyd et al. [45]. SOMDI was employed based on the algorithms described in Lloyd, G. R and Wongravee, K [43,44].

## 3. Results

**Figure 2:**
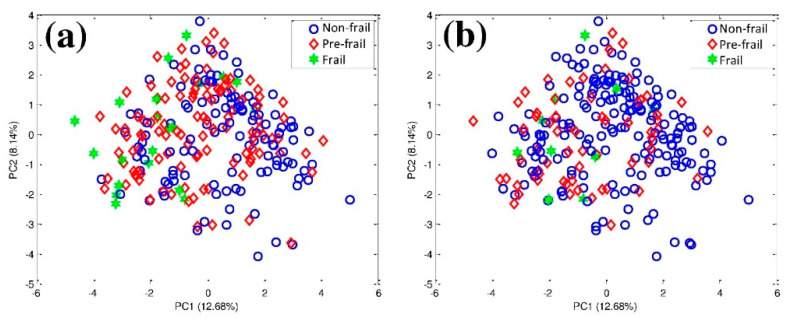
Principal component analysis (PCA) results of the (**a**) modified Fried’s Frailty Phenotype (mFFP), and (**b**) modified Frail Non-Disable (mFiND) questionnaire: **○** Non-frail, ◇ Pre-frail, ✶ Frail.

PCA score plots of each assessment are shown in Figure 2. The frail samples in the mFFP (a) and mFiND (b) questionnaire model are mostly to the left of the non-frail and pre-frail samples. However, they are not completely separated, and there is much overlap between the groups. This implies that the collected data were more complicated than could be illustrated using the linear method. For that reason, the data were further analyzed by SOM, a high-performance non-linear method. 

Table 1 shows the model statistics of the supervised SOM models. Two-thirds of the samples were randomly chosen to be a training set for the models, and one-third were assigned to be a test set for examining the models. The statistic indices, composed of percentage predictive ability (%PA), percentage model stability (%MS), and percentage correctly classified (%CC), were calculated using a bootstrap methodology to evaluate the reliability of the trained models. All the statistic indices showed that the models could properly predict themselves (the trained samples), and that the models of mFiND could provide more than 80% of all indices. Moreover, the mFiND model could predict the unknown samples (the test samples) better than the mFFP model. This implies that the mFiND model is suitable for studying frailty-associated factors in the Thai elderly and, potentially, in other geographic areas.

In this research, the model overfitting problem was avoided by using bootstrap separation. With 50 iterations, two-thirds of the samples were randomly selected and used as training samples, while the rest were used as test samples. However, this sample separation can potentially result in reduced sample numbers. Consequently, the predictive accuracy may not reach the level. A model predictive performance can be improved by increasing the number of pre-frail and frail patients to provide a more representative sample and better predictive results. 

Table 2 shows the discriminant variables’ rank in each classified group. ΔSOMDI values of all variables are shown in Table S2; variables with high ΔSOMDI values were considered influence variables in the group. The personal variables, i.e., income, income source, job before retirement, education, and physical height, were considered to be associated factors for non-frail samples. The disease variables, i.e., cataract/glaucoma, gout and stroke, and the personal variables, i.e., sex, age, polypharmacy, and sufficiency of income, played a crucial role in the pre-frail group. Those variables were also crucial in the frail group, where their importance was indicated by the increased ΔSOMDI of each variable. Cataract/glaucoma, age and sex were the primary variables in the frail group.

**Figure 3:**
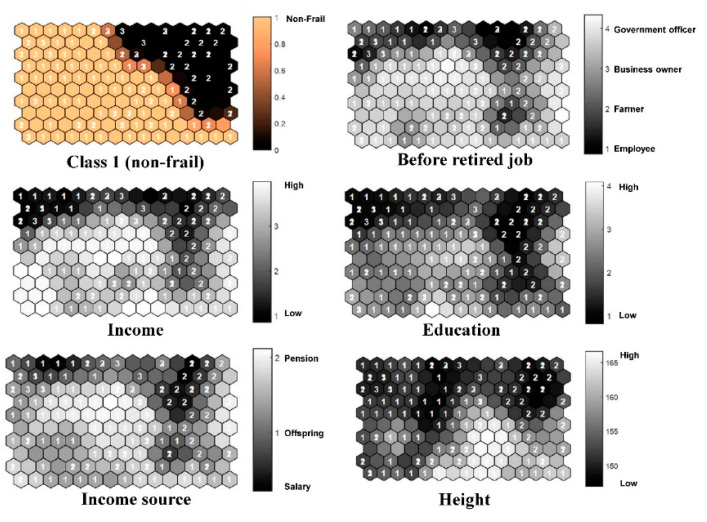
Component planes of the non-frail group and significant variables from the trained SOM model.

Figure 3 shows the component planes of the non-frail group and the significant variables from the trained SOM model. The light and dark copper hexagonal grids represent the frail and non-frail classes, respectively. In the component planes of the significant variables, the light gray grids represent positive results for the diseases and high number values; in contrast, the dark gray grids represent negative results for the diseases and low number values. Most samples in the non-frail group had high income and education levels, and most had been government officers and were currently pensioners. Most of the taller (Height) samples are in the non-frail group.

**Figure 4:**
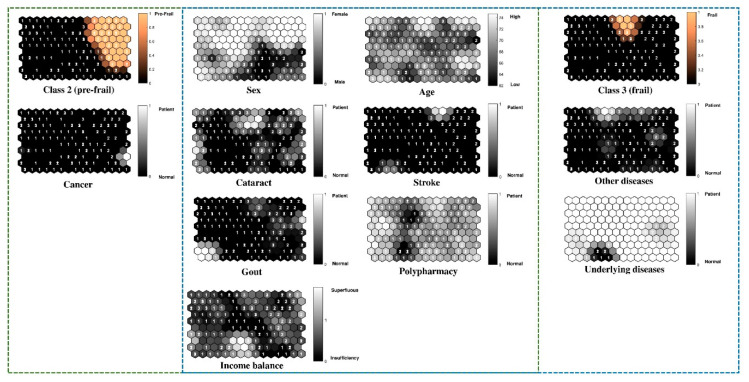
Component planes of the pre-frail and frail groups and their significant variables for the trained SOM model.

Figure 4 shows the component planes of the pre-frail and frail groups and their significant variables for the trained SOM model. The disease variables, i.e., cataract/glaucoma, gout, and stroke, and the personal variables, i.e., sex, age, polypharmacy, and sufficiency of income, were significant in both the pre-frail and frail groups. This implies that most of the samples in the pre-frail and frail group were patients who had cataract/glaucoma, gout, or stroke, and that most were women, elderly, had insufficient income and polypharmacy. In addition, cancer patients are primarily in the pre-frail group, and the majority of patients with other diseases, e.g., myalgia, anemia, dyspepsia, and underlying diseases, e.g., hypertension, diabetes mellitus, dyslipidemia, were in the frail group as well.

## 4. Discussion

The data were analyzed by principal component analysis (PCA), then by supervised self-organizing map (SSOM) using the statistics models %PA, %MS, and %CC to evaluate accuracy. The statistical results (Table 1) show that the model from the mFiND questionnaire had high %PA, %MS, and %CC values, indicating the mFiND questionnaire should be suitable for assessing the variables of each group in the samples. The %CC of the training set was quite high (92.43%), indicating that using the developed SOM model with training samples could match very well with sample class membership. However, the predictive accuracy decreased to 66.53% when the model was tested with unknown samples. This could imply that the model may be prone to overfitting. It also implies that the data could be used for nonlinear classification, where the model iteratively learns from training samples. This overfitting could be avoided by including a greater number of training samples. A component plane can be used to determine how each variable influences the map. By comparing a component plane with the response (class membership) plane of the supervised SOM model, it is possible to investigate their relationship. In this study, the significant variables were identified based on the ranking of the SOMDI values. However, the variation in some parts of the trained component planes may indicate they were not ideally suited for describing the organization of the training samples on the trained map. That variation could, however, simply reflect the complexity of the questionnaire data and indicate that more than one variable should be simultaneously used for determining the level of frailty. 

The significant variables from SOM in the non-frail group (Figure 3) suggest that income and education directly affect health behavior and that higher income and better education can have a shielding effect against frailty. These determinants corresponded with previous research [46], which reported that higher income and social status were linked to better health and that lower education levels were linked to poorer health. Moreover, most of the taller people had a faster walking speed and better walking ability (Figure S1) than other non-frail individuals, indicating that the height variable is important to frailty assessment of the Thai elderly.

According to previous studies, significant variables associated with the pre-frail and frail groups include sex, age, income sufficiency, cataract/glaucoma, gout, stroke and polypharmacy [47,48]. Two of the significant factors related to frailty, sex and age, have been reported to have appeared in virtually all previous studies [49]. This study suggests that women have a higher risk of becoming frail than men, and that the chance of becoming frail increases with age for both genders. A study by Ahmad [50] similarly found that being frail was significantly associated with older age, women, and respondents with a higher number of chronic diseases, poor cognitive function and low socioeconomic status (*p* < 0.05). Although the cancer variable was shown to be significant, there were few samples in this study and all of them were in the same group. Frailty may be an associated factor in cancer patients, e.g., Acosta-Benito MA, et al. [51] found that frailty was a potential prognostic factor in old cancer patients. Other diseases evaluated, e.g., myalgia, anemia, dyspepsia and underlying diseases, e.g., hypertension, diabetes mellitus, dyslipidemia, were also found to be important variables in the frail group. There are, however, other disease factors which could also be associated factors and should be studied further. Variables in the robust, pre-frail and frail groups may be associated with the transition from one state to the next. Stratified and meta-regression analyses in a study by Kojima [52] showed age, gender and follow-up period were associated with frailty transition patterns. Factors associated with frailty transition states should be studied further.

This is the first study to use ANN to analyze frailty-associated factors. The modifiable factors identified as being associated with frailty using this analytical method are reliable, with highly accurate statistical indices, and are in agreement with previous studies. Other variables not included in this study should also be investigated to identify additional factors; ANN should be used to further develop the current model as a frailty assessment tool.

Other underlying diseases not included in this study should be evaluated to determine their association with frailty, including frailty transition states. Additional studies might also be able to identify clinically significant differences in factors associated with frailty in different geographic locations.

One limitation of this study was the use of secondary data, which included a restricted number of variables. A second limitation was that the study methodology reduced the sample numbers, so the predictive accuracy may not reach its maximum level. Further, the model predictive performance could be improved by increasing the number of pre-frail and frail patients to provide a more representative sample, and thus better predictive results.

## 5. Conclusions

The model used in this study can help to identify factors associated with frailty and ageing in Thailand, and, by extension, in other areas of the world. Results from using that model, including assessment of underlying diseases absent from the present study, could potentially encourage the development of methods to prevent and cure modifiable factors associated with frailty, e.g., cataracts/glaucoma, stroke, polypharmacy, and gout. 

## Figures and Tables

**Table 1:** Calculated model statistics of the supervised SOM model.

Assessment	Model Statistic					
	% PA		% MS		% CC	
	Train set	Test set	Train set	Test set	Train set	Test set
mFFP	84.07	50.99	73.75	54.84	90.84	54.58
mFiND	86.67	60.92	80.42	64.65	92.43	66.53

**Table 2:** The significant variables based on change in the self-organizing map discrimination index (ΔSOMDI) values.

Rank	Non-Frail		Pre-Frail		Frail	
	Variables	ΔSOMDI	Variables	ΔSOMDI	Variables	ΔSOMDI
1	Income	0.126	Sex	0.081	CA/GL	0.440
2	Income source	0.093	CA/GL	0.040	Age	0.236
3	JBR	0.093	Gout	0.035	Sex	0.234
4	Educate	0.090	Polypharmacy	0.034	Other diseases	0.171
5	Height	0.079	Stroke	0.032	Stroke	0.112
6			SI	0.030	Polypharmacy	0.097
7			Cancer	0.029	Gout	0.097
8			Age	0.028	UD	0.081
9					SI	0.037

JBR: job before retirement, CA/GL: cataract/glaucoma, SI: sufficiency of income, UD: underlying diseases.

## Supplementary Materials

The following Supplementary Materials are available online at https://www.mdpi.com/1660-4601/17/18/6808/s1, Table S1: Demographic characteristics of elderly participants, Table S2: ΔSOMDI value of each variables responded to mFiND, Figure S1: Height component plane label with a) walk speed of mFFP and b) walking ability of mFiND.
