# Supplementary material for: An Artificial Neural Network Model for Assessing Frailty-Associated Factors in the Thai Population
Source: Int J Environ Res Public Health. 2020 Sep 18;17(18):6808. doi: 10.3390/ijerph17186808 (PMC7558567; doi:10.3390/ijerph17186808)
Supplement: Supplementary file 1 [file ijerph-17-06808-s001.pdf]

Supplementary Materials

Table S1. Demographic characteristics of elderly participants

Characteristic	Number (n = 251)	Percent (%)
1.Sex		
• Male	98	39.04
• Female	153	60.96
2. Age (years)		
• 60-69	164	65.34
• 70-79	71	28.29
• >80	16	6.37
3. Marital Status		
• Married	176	70.12
• Divorced	58	22.71
• Single	14	5.58
• Separated	4	1.59
4. Level of education		
• Less than high school	79	31.48
• High school	72	28.69
• Higher than high school	71	28.29
• Other degree	28	11.16
5. Religion		
• Buddhist	243	96.81
• Christian	8	3.19
6. Occupation (before retirement)		
• Government officer	127	50.60
• Business owner	63	25.00
• Farmer	23	9.16
• Employee	19	7.57
• Other	19	7.57
7. Monthly income (Thai baht)		
• > 10,000	129	51.39
• 5,001 – 10,000	30	11.95
• 1,000 – 5,000	29	11.95
• < 1,000	63	25.10
8. Sources of income		
• Pension	150	59.76
• Offspring	55	21.91
• Salary	46	18.33

9. Income sufficiency		
• Sufficient	170	67.73
• Insufficient	53	21.12
• More than sufficient	28	11.16
10. Medical expenses payer		
• Government	223	88.84
• Social welfare	8	3.19
• Individual	20	7.97
11.Body mass index (BMI) (kg/m^2)		
• < 20.00	17	6.77
• 20.00 – 24.99	98	39.04
• 25.00 – 29.99	112	44.63
• ≥ 30.00	24	9.56
12. Underlying disease		
• Yes	232	92.43
• No	19	7.57
13. List of diseases		
• Hypertension	165	65.73
• Dyslipidemia	79	31.46
• Diabetes mellitus	42	16.72
• Gout / osteoarthritis	26	10.35
• Glaucoma /cataract	23	9.16
• Chronic kidney disease	19	7.56
• Benign prostatic hypertrophy	11	4.38
• Coronary artery disease	8	3.18
• Cerebrovascular disease	5	1.98
• Cancer	2	0.78
• Others	22	8.76
14. Number of medications		
• 0-1	39	15.53
• 2-3	127	50.59
• > 3	85	33.86

Most of the participants (Table 1) were female (60.96%), 60-69 years (65.34%), married (70.12%), had less than a high school education (31.48%), were Buddhist (96.81%), had a monthly income >10,000 Thai baht (51.39%), received their income from a pension (59.76%), reported their income was sufficient (67.73%), had their medical expense paid by the government (88.84%),

had an underlying disease (92.43%), had hypertension (65.73%) and used 2-3 medications (50.59%)

Table S2. ΔSOMDI values of variables in the mFiND models

Variables	Delta-z		
	Non-frail	Pre-frail	Frail
Sex	-0.068	0.081	0.234
Age	-0.042	0.028	0.236
Marital status	-0.010	0.024	-0.017
Religion	-0.004	0.017	-0.038
Education	0.090	-0.168	-0.063
Job before retirement	0.093	-0.135	-0.219
Job after retired job	0.029	-0.046	-0.052
Income level	0.126	-0.203	-0.218
Income source	0.093	-0.167	-0.095
Sufficiency of income	-0.019	0.030	0.037
Body weight	0.027	-0.034	-0.085
pre-body weight	0.013	-0.016	-0.045
Weight change	0.049	-0.064	-0.145
Height	0.079	-0.116	-0.189
Receiving medical treatment	-0.014	0.025	0.010
Underlying disease	-0.017	0.015	0.081
Stroke	-0.029	0.032	0.112
Myocardial infarction	0.031	-0.051	-0.046
Gout	-0.029	0.035	0.097
Cataract	-0.073	0.040	0.440
Cancer	-0.012	0.029	-0.018
Hypertension	-0.010	0.026	-0.024
Chronic kidney diseases	0.016	-0.024	-0.037
Diabetes Mellitus	-0.002	0.023	-0.077
Benign prostatic hyperplasia	0.002	0.011	-0.060
Dyslipidemia	0.035	-0.045	-0.109
Other diseases	-0.032	0.022	0.171
Polypharmacy	-0.028	0.034	0.097

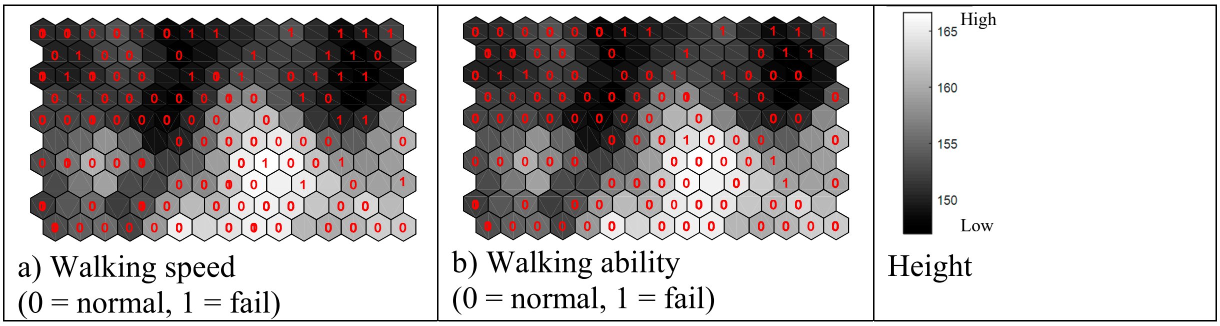

Figure S1. Height component plane label with a) walking speed from mFFP and b) walking ability from mFiND

Walking speed/walking ability in the height component plane illustrates that taller people generally have a faster walking speed and better walking ability than shorter non-frail individuals.
